# Use of Exogenous Testosterone for the Treatment of Male Factor Infertility: A Survey of Nigerian Doctors

**DOI:** 10.1155/2017/4607623

**Published:** 2017-08-29

**Authors:** Olufunmilade Akinfolarin Omisanjo, Stephen Odunayo Ikuerowo, Moruf Adekunle Abdulsalam, Sheriff Olabode Ajenifuja, Khadijah Adebisi Shittu

**Affiliations:** ^1^Department of Surgery, Lagos State University College of Medicine and Teaching Hospital (LASUCOM/LASUTH), Ikeja, Lagos, Nigeria; ^2^Department of Surgery, Lagos State University Teaching Hospital (LASUTH), Ikeja, Lagos, Nigeria; ^3^Department of Obstetrics and Gynaecology, Lagos State University Teaching Hospital (LASUTH), Ikeja, Lagos, Nigeria

## Abstract

**Background:**

Though exogenous testosterone is known for its contraceptive effects in men, it is sometimes prescribed by medical practitioners for the treatment of male factor infertility in the mistaken belief that exogenous testosterone improves sperm count. The aim of this study was to evaluate the scope of testosterone use in the treatment of male factor infertility by medical practitioners in Lagos, Nigeria.

**Methods:**

A survey using a structured questionnaire was carried out amongst doctors attending a regular Continuing Medical Education (CME) programme in Lagos, Nigeria.

**Results:**

There were 225 respondents. Most of the respondents (69.8%, *n* = 157) indicated that exogenous testosterone increases sperm count. Only 22 respondents (9.8%) indicated (correctly) that exogenous testosterone decreases sperm count. Seventy-seven respondents (34.2%) had prescribed some form of exogenous testosterone in the treatment of male factor infertility. The vast majority of respondents who had prescribed testosterone (81.8%, *n* = 63) thought exogenous testosterone increases sperm count. There was no statistically significant difference in the pattern of prescription across the respondents' specialty (*p* = 0.859) or practice type (*p* = 0.747).

**Conclusion:**

The misuse of exogenous testosterone for the treatment of male infertility was common amongst the respondents, with most of them wrongly believing that exogenous testosterone increases sperm count.

## 1. Introduction

The World Health Organization defines infertility as the inability of a sexually active, noncontracepting couple to achieve spontaneous pregnancy in one year [1]. Infertility affects approximately 15% of couples [2]. The male factor is estimated to be solely responsible in up to 20% of affected cases, with an additional 30–40% of cases involving both male and female factors [3, 4].

Causes of male infertility vary and include congenital or acquired abnormalities, immunological factors, malignancies, urogenital tract infections, endocrine disturbances, and the presence of varicoceles [1, 5].

There are different treatment options for male factor infertility and these may range from lifestyle modification, use of various combinations of medications, and hormones, for example, clomiphene citrate and human chorionic gonadotrophin, to surgical procedures like varicocele ligation and orchidopexy [6]. The advent of assisted reproduction has revolutionized the treatment of infertility leading to the setting up of dedicated fertility centers.

While some of the couples with infertility are treated in these established fertility centers, a sizeable number of them get their initial evaluation and even treatment started by general practitioners and gynaecologists.

The dearth of urologists in underdeveloped countries also means that most of the couples with male factor infertility are treated by gynaecologists and general practitioners without any urology review in countries like Nigeria.

It is not unusual for some male patients with infertility to present with features of hypogonadism. While exogenous testosterone may be useful in the alleviation of some of the symptoms of hypogonadism in such males, its use leads to reduction in sperm count [7]. It is therefore not recommended that exogenous testosterone be used alone in the treatment of male factor infertility even when there are associated features of hypogonadism.

In spite of the above, it is not uncommon for doctors to place patients with male factor infertility on testosterone alone in the mistaken belief that administration of exogenous testosterone improves sperm count.

Samplaski et al. found that 1.3% of 4400 patients in their male infertility database were on exogenous testosterone and the majority of these patients had their therapy prescribed to them by endocrinologists and family practitioners [8].

In 2012, Ko et al. in a survey of the American Urological Association (AUA) had expressed concern that 25% of the respondents in a survey of urologists use exogenous testosterone, a medication known for its contraceptive potential for male infertility treatment [9].

There are no reports in Nigeria on the pattern of use of exogenous testosterone in the treatment of male infertility, though a review of the referrals to our urology clinic over the years suggests that this practice is not uncommon.

The aim of our study was therefore to document the pattern of use of exogenous testosterone in the treatment of male infertility by doctors in Lagos, Nigeria.

## 2. Materials and Methods

A survey was carried out amongst doctors attending a regular Continuing Medical Education (CME) programme at the Lagos State University Teaching Hospital (LASUTH), Ikeja, Lagos. The CME which was under the auspices of the Association of General and Private Medical Practitioners of Nigeria (AGPMPN) included broad topics across general practice and was open to doctors from different backgrounds.

A structured questionnaire was used.

Data were captured with Microsoft Excel and data analysis was done with the Statistical Package for Social Sciences (SPSS version 21).

Comparison of means was performed using Student's* t*-test while Fischer's exact test was used for contingency table analysis with a two-tailed *p* value of <0.05 accepted as being statistically significant.

## 3. Results

There were 225 respondents available for analysis out of the 241 questionnaires administered (93.4% response rate).

One hundred and eighty-six (82.7%) were general practitioners and 38 (16.9%) were gynaecologists. One respondent did not indicate his specialty (Table 1).

One hundred and forty-two respondents (63.15%) were in public service and 81 (36%) were in private practice. Two (0.9%) respondents were in other types of practice.

Most of the respondents (69.8%, *n* = 157) indicated that exogenous testosterone increases sperm count, 4.9% (*n* = 11) indicated that it had no effect, and 12.9% (*n* = 29) were unsure of the effect of testosterone on sperm count. Only 22 respondents (9.8%) indicated (correctly) that exogenous testosterone decreases sperm count (Table 2).

Seventy-seven respondents (34.2%) had prescribed some form of exogenous testosterone for the treatment of male factor infertility.

Most of the respondents who had prescribed testosterone were general practitioners (85.7%, *n* = 66).

Amongst the respondents who had prescribed testosterone, 81.8% (*n* = 63) thought exogenous testosterone increases sperm count.

Parenteral testosterone was the commonest form of testosterone prescribed (67.5%, *n* = 52). Other testosterone preparations prescribed were as follows: oral (*n* = 22, 28.6%), gel (*n* = 1, 1.3%), other unspecified forms (*n* = 1, 1.3%), and form not indicated (*n* = 1, 1.3%) (Table 3). There was no statistically significant difference in the pattern of prescription across the respondents' specialty (*p* = 0.859) or practice type (*p* = 0.747).

While a pretreatment seminal fluid analysis (SFA) was obtained by most of the respondents (90.9%, *n* = 70), a pretreatment testosterone was only done in 62.3% (*n* = 48). There was no statically significant difference across specialty (*p* = 0.012) or practice type (*p* = 0.054).

A posttreatment SFA was obtained by 83.1% of the respondents (*n* = 64), while a posttreatment testosterone was done by only 41.6% of the respondents (*n* = 32).

Most of the respondents (61.8%, *n* = 139) indicated that male factor infertility should be treated by urologists (Table 4). One hundred of the respondents (70.4%) in public practice as against 48.1% (*n* = 39) of those in private practice had indicated that treatment should be by the urologist, and this was statistically significant (*p* = 0.001).

## 4. Discussion

Infertility can be quite a distressing medical condition. Proper identification of the aetiology and appropriate early treatment are vital.

The use of any treatment that may worsen the condition is definitely unwelcome and that is why exogenous testosterone alone should not be used in the treatment of male factor infertility.

Several studies have shown the contraceptive potential of exogenous testosterone [10–13].

Exogenous testosterone given alone reduces intratesticular testosterone and spermatogenesis with consequent oligospermia and sometimes azoospermia [7, 14–17].

Bang et al. had documented that the misuse of exogenous androgen replacement therapy in infertile men with poor sexual function can cause temporary spermatogenic dysfunction, thus aggravating infertility [18].

Though clinical experience suggests that the use of testosterone for the treatment of male factor infertility may be commonplace in our environment, our study documents for the first time the scope of this practice amongst medical practitioners in Nigeria.

The knowledge of the correct effect of testosterone on sperm count was poor amongst the respondents with only 9.8% of them indicating the correct effect. Though disturbing, this was not unsurprising given that most of the respondents were general practitioners who ordinarily are not specialized in male fertility treatment.

The use of testosterone was commonplace (34.2%). Most of the use (85.7%) was by general practitioners in keeping with the findings of Samplaski et al. [8]. The level of prescription which we found amongst our respondents (34.2%) was comparable to the 25% reported amongst urologists by Ko et al. in the USA [9]. These high figures suggest that the problem of the wrong use of testosterone is not limited to developing countries. National urological and medical societies need to survey their respective local practices as the problem may be more widespread than expected.

The major reason for the use of exogenous testosterone for the treatment of male factor infertility is the mistaken belief that testosterone increases sperm count as the vast majority of respondents who had prescribed testosterone (81.8%, *n* = 63) had indicated that exogenous testosterone increases sperm count. Educating all doctors involved in the management of men with infertility on the correct effect of exogenous testosterone on spermatogenesis is important to address this misconception. This is especially important for general practitioners who invariably are the ones who initially manage most of these patients because of the paucity of urologists in our environment.

A substantial number of the patients were commenced on the testosterone treatment without a justifiable indication as a testosterone assay was done by only 62.3% of the respondents who had administered testosterone.

Though some male patients with infertility may also have hypogonadism, the use of hCG, clomiphene citrate, and anastrozole has been shown to be safe and efficacious alternatives to testosterone and is therefore preferred for the initiation and maintenance of spermatogenesis in these patients [19–21].

Almost a fifth (16.9%) of the respondents did not assess the sperm count after commencement of exogenous testosterone. Monitoring posttreatment seminal fluid parameters is important because this can alert the physician to the deteriorating sperm count and the need for discontinuation of the testosterone injection. This early discontinuation is important because though the suppression of spermatogenesis by exogenous testosterone is considered reversible following cessation of treatment, in some instances, this suppressive effect on spermatogenesis may persist as shown by Kolettis et al. [22].

It was curious that the respondents had initiated the treatment for male factor infertility as a sizeable number of them (61.8%)—though only significantly those in public practice—had indicated that urologists should be the ones to treat male factor infertility.

## 5. Conclusion

The misuse of testosterone in the treatment of male infertility was common amongst the respondents and this stemmed from a poor knowledge of the correct effect of exogenous testosterone on sperm count.

There is need for better education of doctors especially general practitioners. Emphasis also has to be placed on the need for early referral of couples with male factor infertility for timely urological evaluation and necessary treatment as male factor infertility might be too complex to be handled by general practitioners who already have their hands full treating other ailments.

## Figures and Tables

**Table 1:** Specialty of respondents.

Specialty	Frequency	Percentage (%)
General practitioner	186	82.7
Gynaecologist	38	16.9
Others	1	0.4

Total	225	100

**Table 2:** Indicated effect of exogenous testosterone on sperm count (SC).

Indicated effect	Frequency	Percentage (%)
Increases SC	157	69.8
Decreases SC	22	9.8
No effect on SC	11	4.9
Not sure of effect on SC	29	12.9
No response	6	2.7

Total	225	100

**Table 3:** Form of testosterone prescribed.

Testosterone preparation	Frequency	Percentage (%)
Parenteral	52	67.5
Oral	22	28.6
Gel	1	1.3
Others	1	1.3
No response	1	1.3

Total	77	100

**Table 4:** Respondents' indicated response to “who should treat male factor infertility (MFI)?”

Who should treat MFI?	Frequency	Percentage (%)
General practitioner	17	7.6
Gynaecologist	51	22.7
Urologist	139	61.8
No response indicated	18	8

Total	225	100

## Appendix

### Copy of the Questionnaire

*Use of Testosterone in the Treatment of Male Infertility*. We are conducting a study on the use of Testosterone Supplement in the treatment of Male factor Infertility by doctors. We will be grateful if you can kindly spare 5 minutes to fill this questionnaire.

Thank you.

1. You practise as a:
  - General Practitioner
  - Gynaecologist
  - Urologist
  - Others
2. Your practice is primarily:
  - Public based
  - Private based
  - Others
3. Exogenous Testosterone does the following to Sperm Count:
  - Increases Sperm Count
  - Decreases Sperm Count
  - Has no effect on Sperm Count
  - Not sure
4. Have you used any form of Exogenous Testosterone to treat men with Male factor Infertility?
  - Yes
  - No
  - (If you answered NO to Question (4), you may skip to Question (10).
  - If you answered YES to Question (4), kindly continue with the remaining Questions (5)–(10))
5. What form of testosterone did you use? (Tick all that is applicable)
  - Intramuscular/Parenteral
  - Oral
  - Gel
  - Others
6. Did you check the pretreatment Testosterone?
  - Yes
  - No
7. Did you do a pretreatment Seminal Fluid Analysis (SFA)?
  - Yes
  - No
8. Did you monitor the testosterone level while the patient was on treatment?
  - Yes
  - No
9. Did you do any Post-treatment Seminal Fluid Analysis?
  - Yes
  - No
10. Who should treat Male factor Infertility?
  - General Practitioners
  - Gynaecologists
  - Urologists

Thank you for sparing your precious time.

Dr O. A. Omisanjo

(Dept of Surgery, LASUTH/LASUCOM, Ikeja, Lagos.)
